# Cognitive behavioural therapy attenuates the enhanced early facial stimuli processing in social anxiety disorders: an ERP investigation

**DOI:** 10.1186/s12993-017-0130-7

**Published:** 2017-07-28

**Authors:** Jianqin Cao, Quanying Liu, Yang Li, Jun Yang, Ruolei Gu, Jin Liang, Yanyan Qi, Haiyan Wu, Xun Liu

**Affiliations:** 1 Department of Nursing, Harbin Medical University, Daqing, Heilongjiang Province China; 2 Laboratory of Movement Control and Neuroplasticity, KU Leuven, 3001 Louvain, Belgium; 3 Neural Control of Movement Laboratory, ETH Zurich, 8057 Zurich, Switzerland; 4 CAS Key Laboratory of Behavioral Science, Institute of Psychology, Chinese Academy of Sciences, 16 Lincui Road, Chaoyang District, Beijing, 100101 China; 5 Department of Psychology, University of Chinese Academy of Sciences, Beijing, 100101 China

**Keywords:** Social anxiety disorder, Cognitive-behavioural therapy, P1, N170, Hyper-vigilance

## Abstract

**Background:**

Previous studies of patients with social anxiety have demonstrated abnormal early processing of facial stimuli in social contexts. In other words, patients with social anxiety disorder (SAD) tend to exhibit enhanced early facial processing when compared to healthy controls. Few studies have examined the temporal electrophysiological event-related potential (ERP)-indexed profiles when an individual with SAD compares faces to objects in SAD. Systematic comparisons of ERPs to facial/object stimuli before and after therapy are also lacking. We used a passive visual detection paradigm with upright and inverted faces/objects, which are known to elicit early P1 and N170 components, to study abnormal early face processing and subsequent improvements in this measure in patients with SAD.

**Methods:**

Seventeen patients with SAD and 17 matched control participants performed a passive visual detection paradigm task while undergoing EEG. The healthy controls were compared to patients with SAD pre-therapy to test the hypothesis that patients with SAD have early hypervigilance to facial cues. We compared patients with SAD before and after therapy to test the hypothesis that the early hypervigilance to facial cues in patients with SAD can be alleviated.

**Results:**

Compared to healthy control (HC) participants, patients with SAD had more robust P1–N170 slope but no amplitude effects in response to both upright and inverted faces and objects. Interestingly, we found that patients with SAD had reduced P1 responses to all objects and faces after therapy, but had selectively reduced N170 responses to faces, and especially inverted faces. Interestingly, the slope from P1 to N170 in patients with SAD was flatter post-therapy than pre-therapy. Furthermore, the amplitude of N170 evoked by the facial stimuli was correlated with scores on the interaction anxiousness scale (IAS) after therapy.

**Conclusions:**

Our results did not provide electrophysiological support for the early hypervigilance hypothesis in SAD to faces, but confirm that cognitive-behavioural therapy can reduce the early visual processing of faces. These findings have potentially important therapeutic implications in the assessment and treatment of social anxiety.

*Trial registration* HEBDQ2014021

## Background

Social anxiety disorder (SAD) is a mental disorder characterized by significant fear of negative evaluation and avoidance of interpersonal situations [1, 2]. Over the past decades, an extensive body of research has been devoted to various cognitive symptoms related to SAD, such as attentional biases, negative interpretation biases, and expectancy and memory biases [3–10]. Cognitive models of anxiety [11, 12] have suggested that information processing biases lead patients with social anxiety to view social situations in an excessively negative fashion [13–15]. Due to this interpretation bias, patients with SAD tend to judge ambiguous faces as more angry than happy [16]. However, it is worth mentioning that another study did not report similar interpretation bias [17]. If such interpretation bias exists in SAD for faces, even neutral faces may be viewed as more negative by patients with SAD. Considering that many behavioural and neuroimaging studies have provided convincing evidence that social anxiety is linked to attention bias toward threatening facial stimuli [18–23], there may also be enhanced attention toward neutral faces when compared to those of healthy control subjects.

Compared to other objects, the human face is prominent due to its capacity to convey attractiveness, trustworthiness, or emotions with biological significance in social interactions [24–27]. Studies have indicated that faces with direct eye contact may elicit avoidant and escape responses in individuals with social anxiety disorders [28, 29]. For example, a study using a modified dot-probe task (objects vs. expressions) showed that individuals with social phobia direct their attention away from faces and toward household objects [28]. This suggests that individuals with social anxiety may have impaired processing of faces. Although much effort has been made to examine attention bias or processing abnormalities in response to different emotional expressions in individuals with social anxiety, to our knowledge, no study has directly investigated facial perception abnormalities per se in comparison to object perception in patients with SAD. Therefore, whether SAD is associated with abnormal early processing of facial stimuli remains unclear.

Electrophysiological brain responses may be useful in clarifying whether SAD is associated with enhanced early processing of facial stimuli, as they have high temporal resolution, which may help to differentiate early vs. late attention processes during the processing of facial stimuli. Previous studies have identified different ERP components reflective of different stages of facial perception or attention bias [30–35]. It has been demonstrated that threat-related faces elicit shorter latencies and greater amplitudes of early ERP components (e.g., the P1, N1, and N170) in individuals with high anxiety than in those with low anxiety [30, 36]. An ERP study used the Stroop paradigm with non-emotional stimuli, explicit emotion tasks, and implicit emotion tasks to examine the impact of perceptual and task factors on facial processing in individuals with social anxiety. The authors found that there were enhanced P1 responses during all tasks in individuals with social anxiety, and that this effect was independent of the effects of perceptual or task factors [37]. Such P1 enhancement effects were also found in individuals with sub-clinical SAD in response to happy, angry, fearful, disgusted, and neutral faces. This indicates that there is early attentional capture even by neutral faces in individuals with SAD [38].

Another face-sensitive ERP component is the N170, which is a temporal-parietal negativity associated with facial perceptual coding [39–44]. Prior studies have indicated that N170 is enhanced in response to inverted faces (N170 face inversion effect), but not inverted objects. This supports the idea that N170 is specifically affected by faces [44–46]. The processing of inverted faces typically involves additional early recruitment of visual processing resources when compared to that of upright faces [47]. Social anxiety has been characterized by attentional bias toward threatening or ambiguous faces. However, the manner in which social anxiety affects the processing of inverted faces and whether therapy can alter such processing abnormalities remain unclear.

Cognitive-behavioural therapy (CBT) is a time-limited present-oriented approach to psychotherapy that teaches patients the cognitive and behavioural competencies required to function adaptively in their interpersonal and intrapersonal worlds. CBT is also the most studied non-pharmacologic approach for the treatment of social anxiety disorder [48]. To date, a large number of investigations have demonstrated the efficacy of CBT as a treatment for SAD [49–53]; Scaini et al. [54, 55]. Cognitive-behavioural group therapy (CBGT) has also been reported to be an effective therapeutic approach in reducing the symptoms of anxiety and depression in individuals with anxiety disorders [56–59]. For instance, using an Educational Supportive Group Psychotherapy (ESGP) group as a control, Heimberg et al. [60] examined the effectiveness of CBGT in SAD. They found that the phobic severity rating scale scores of patients undergoing CBGT improved to a great extent after treatment. These patients also reported less anxiety before and during the behavioural test. Interestingly, the 5-year follow-up study (patients who received CBGT or an alternative treatment were contacted 4.5–6.25 years after the initial treatment) also suggested that patients who received CBGT had more lasting improvements than those receiving ESGP treatments [61]. Few studies regarding the treatment of SAD have investigated neuroimaging data or brain activity changes pre- and post-therapy [62–65].

A handful of studies reported in the literature suggest that therapy leads to global modulations of brain activity, such as attenuated amygdala activity [65]. However, studies of EEG data on therapy effect in patients with SAD are rare. To our knowledge, there are two EEG studies have examined such therapy induced EEG change effect on SAD. One study indicated patients shifted significantly from greater relative right to greater relative left resting frontal EEG activity from pre- to post treatment [66], while another suggested greater coupling between EEG delta and beta oscillations in pre-treatment SAD than control and the coupling EEG normalized after treatment [67]. Compared to these EEG in rest studies, event-related potential(ERP) technique, which permit to investigate behavioural and neural activity(specific ERP components) and relationship between the two with high temporal resolution, may contribute to testing our hypothesis of the underlying early face or object processing mechanism of abnormality in SADs.

Despite these advances, clinical studies have yet to examine whether SAD is associated with abnormal early processing of facial stimuli and whether CBGT can reduce such abnormal neural symptoms. The primary objective of this study was to examine whether social anxiety is associated with abnormalities at the early stages of processing of face-related information, and to investigate neural changes (i.e., P1–N170 effect) after CBGT. The P1–N170 effect, include the P1 effect, the N170 effect and the slope from P1 to N170. Based on the hypervigilance hypothesis of SAD, we expected to observe an enhanced P1–N170 effect in SAD patients. That is, we hypothesized that SAD will show larger P1 and N170 amplitude and larger P1–N170 slope when they processing face-configurational information, especially for inverted faces. Furthermore, we also expect a treatment effect on theses enhanced early P1/N170 or the P1–N170 slope effect. Specifically, after CBGT, we expected to find decreased P1–N170 responses to faces, which would be reflective of improvements in anxiety symptoms in patients with SAD.

## Methods

### Participants

The study was carried out in accordance with the Declaration of Helsinki and the experimental protocols used were approved by the institutional review board (IRB) of Harbin Medical University. Eighteen outpatients with SAD were recruited from the Psychology Department of Affiliated Hospital of Harbin Medical University, while 18 healthy control (HC) participants were recruited through advertisements. The patients with SAD (3 men and 15 women, mean age = 33.61 ± 8.84 years) were diagnosed using the validated Chinese translation of the Structured Clinical Interview for Diagnostic and Statistical Manual of Mental Disorders, Fourth Edition (DSM-IV) (SCID-IV) [68], which is the gold standard for assessing SAD in China. Control participants were demographically matched HCs with no history of DSM-IV psychiatric disorders. Specifically, participants’ gender, age and education year are being matched and detail information for both groups are shown in Table 1. These individuals were also screened using the SCID. All participants were right-handed and reported no psychoactive substance abuse, no unstable medical illness, and no past or current neurological illness. The subjects’ anxiety symptoms were assessed using the brief social phobia scale (BSPS, [69]) and the interaction anxiousness scale (IAS, [70]). All participants provided written informed consent for the experiment. One patient with SAD and the matched HC were excluded because they dropped out from the therapy. We thus carried out the study using a sample of 17 patients with SAD and 17 HC participants. Demographic data and the self-reported measures of the final 34 participants in the two groups are presented in Table 1. As shown in Table 1, the groups did not differ in demographic characteristics. Compared to those in the HC group, participants in the SAD group reported higher levels of social anxiety.

**Table 1:** Summary of sociodemographic and self-report measures of mood and symptom severity for participants with social anxiety disorder (SAD) and healthy controls

	SAD group (n = 17)	Healthy controls (n = 17)	*T or* χ^2^-test (*df* = 32)
Age (years)	33.29 (9.01)	33.65 (9.42)	−0.112
Sex (% women)	82%	82%	
Education (years)	13.24 (2.05)	14.29 (2.17)	−1.46
IAS	57.59 (5.23)	34.47 (6.97)	10.932^***^
BSPS	45.12 (11.51)	9.65 (5.43)	11.491^***^
Flower counts	Pre: 60.13; post: 60	Pre: 59.82; post: 59.88	All *p* > 0.19

Values provided as means (standard deviations)

*SAD* social anxiety disorder, *IAS* interaction anxiousness scale, *BSPS* brief social phobia scale

*** *p* < 0.001

### Stimuli and passive visual detection paradigm

**Fig. 1:**
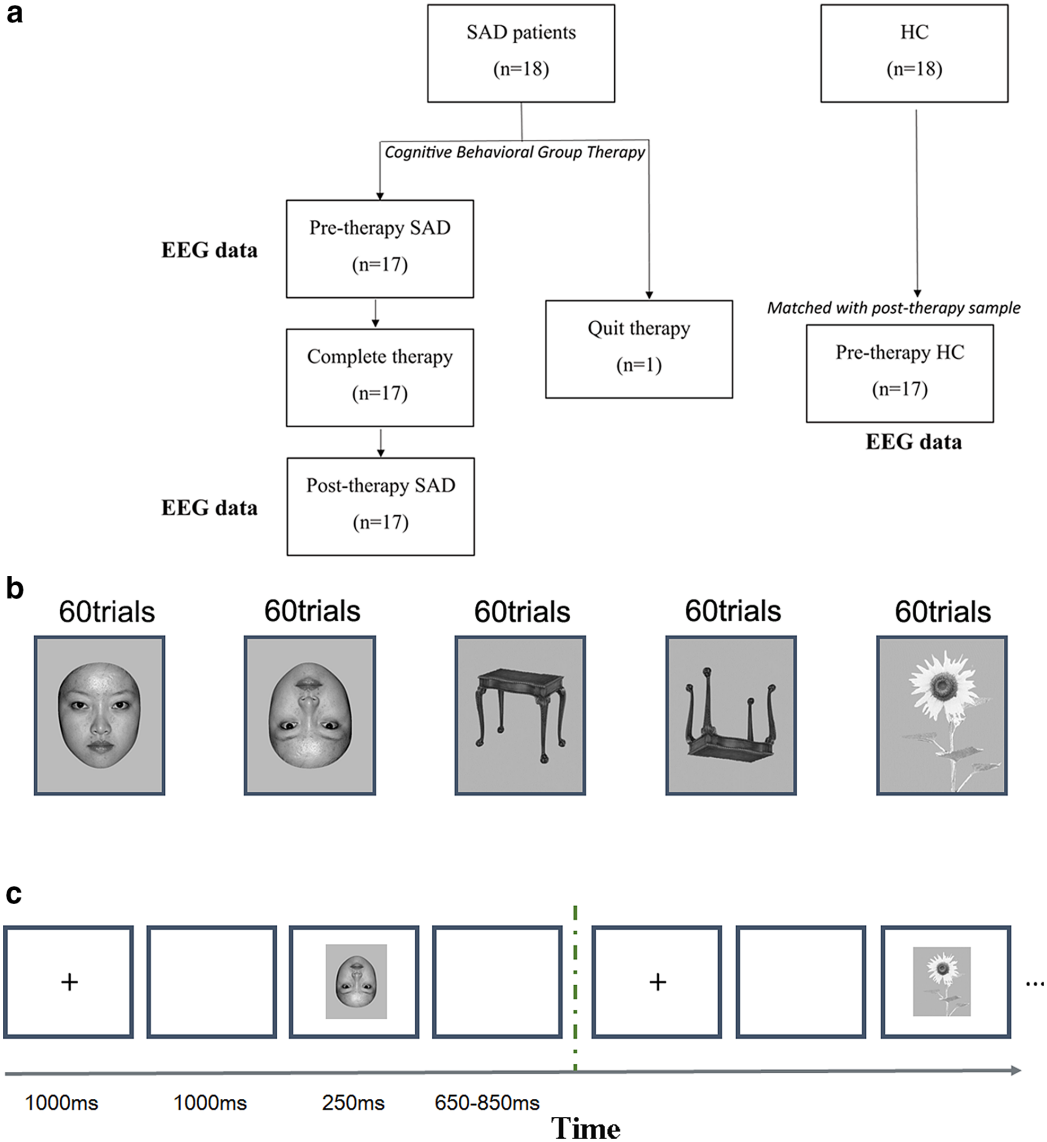
**a** The scheme of the data and samples study; **b** examples of face stimuli (upright and inverted), objects (upright and inverted), and target flowers used in our experiments; **c** schematic examples of trials used in each block. The block began with the presentation of a cross for 1000 ms. This was followed by 950–1050 ms of a blank screen and a sequence of 100 trials. Every block comprised the presentation of *upright faces*, *upright tables*, *inverted faces*, *inverted tables*, and targets. There were 20 trials in total. All stimuli were presented randomly with 250-ms durations and an inter-stimulus intervals randomized to range from 650 to 850 ms. Participants were asked to focus on the centre of the screen, to count the number of the target flowers in their minds, and to ignore other stimuli. At the end of each block, the subjects reported the number of flowers they had counted

The paradigm was adopted from He et al. [71]. The stimuli used in the study included 60 photographs of unfamiliar young faces, 60 photographs of tables, and 60 photographs of flowers, which were all selected from neutral pictures in the Chinese affective picture system (CAPS). Pictures were all in grey-scale and all were resized to 8 cm × 12 cm, half of the faces were of men and the other half were of women. All face stimuli were trimmed to exclude hair and non-facial contours (Fig. 1). There are five stimulus conditions in the passive visual detection paradigm: upright faces, inverted faces, upright tables, inverted tables, and upright flowers. Sixty trials for each condition were presented in the EEG experiment (total of 300 trials). The stimuli were presented at the centre of a computer screen and were viewed from a distance of 80 cm. All stimuli were presented on a blank background shown on a 17-in. computer screen using a personal computer running E-Prime.

Each trial began with the presentation of a stimulus cross for 1000 ms, followed by a blank screen of 950–1050 ms. All stimuli were presented randomly with 250-ms durations and inter-stimulus intervals randomized to range from 650 ms to 850 ms. Subjects were instructed to pay attention to the presented stimuli and to count the flowers (i.e., targets) in each presentation block. The subjects reported the number of flowers counted upon completion of the viewing of each block. All stimuli were randomized and counterbalanced across participants. Every block comprised of upright faces, upright tables, inverted faces, inverted tables, and targets, 20 trials of each condition in one block. There were 60 trials for each condition presented in three blocks.

### Cognitive behavioural group therapy

CBGT was administered by a clinical psychologist and a college psychology teacher. The therapy comprises intensive intervention and consolidation of treatment for 37.5 h over 1 year. The CBGT procedure was conducted following the CBGT treatment guidelines established in Heimberg et al. [72], which includes (1) psychological education, (2) assessment of conceptual ability, (3) cognitive modules, and (4) a behaviour module. The intensive intervention was performed over 12 sessions and lasted 30 h in total (4 sessions/month, lasting 3 months). In the first three sessions, psychological education and assessment of conceptual ability are conducted to achieve case conceptualization. Patients were taught to understand the normalization and change laws of anxiety, the cognitive model of SAD, factors responsible for the maintenance or an increase in anxiety, and so on. These sessions were designed to enable patients to identify negative cognition (“automatic thoughts” [ATs]), to observe covariation in anxiety, to understand ATs and behavioural responses, to set treatment goals, to master completing homework, etc. The cognitive module and behavioural module are presented in the 4th to 11th sessions. In these sessions, patients are taught to use disputation, coping skills, behavioural experiments, exposure skills, and role-playing to challenge logical errors in their ATs. They are also taught to formulate rational alternatives and behavioural responses. Furthermore, they confront increasingly difficult feared situations (in the session and in real life) while applying cognitive and behavioural skills. When the patients worked on their personal target situations, a standard sequence was followed: identification of ATs and identification of logical errors in ATs, which was followed by disputation of ATs and formulation of rational responses. Thereafter, patients practiced cognitive skills while completing behavioural tasks (e.g., conversing with another group member or giving a speech). Goal attainment and use of cognitive skills were reviewed. Behavioural experiments were used to confront specific reactions to the exposure. Patients were provided with assignments pertinent to exposure to real-life situations across the sessions. In the 12th session, patients were instructed to complete self-administered cognitive restructuring and behavioural skill exercises in real life.

The consolidation treatment is carried out to ensure that the patients implement the self-administered cognitive restructuring and behavioural skill exercises in real life. According to the patient’s feedback, the researcher and the intervener provide further guidance for better therapeutic effect. Consolidation treatments were implemented after the 3rd, 6th, and 9th months of the intensive intervention for 7.5 h. The therapy session lasted for about 1 year and the time interval between the two EEG recordings in patients with SAD was 1 year. Patients with SAD performed the passive visual detection paradigm before and after the therapy.

### EEG recording and pre-processing

The participants sat comfortably in an electrically shielded room approximately 80 cm from a computer screen. The EEG data were recorded using a 64-channel NeuroScan system (NeuroScan Inc., Herndon, VA). Raw EEG data were sampled at 1000 Hz/channel, with impedances lower than 5 kΩ. Vertical electrooculograms (VEOGs) were recorded supra- and infra-orbitally at the left eye. Horizontal electrooculograms (HEOGs) were recorded by electrodes at the left and right orbital rims. Online recordings were referenced to the nasion. We used a bandpass filter of 0.05–100 Hz.

**Fig. 2:**
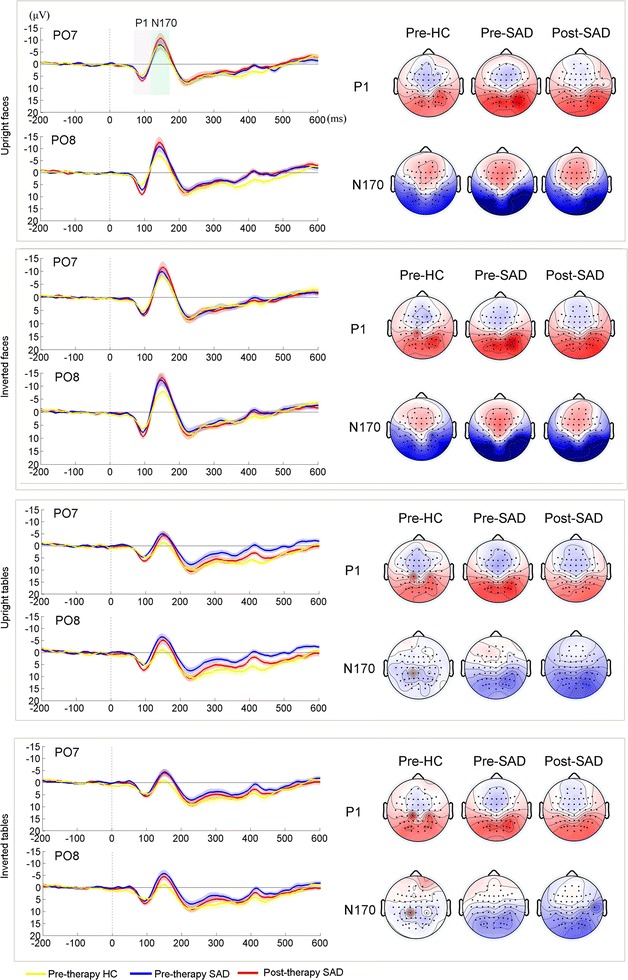
P1 and N170 analyses for *upright faces*, *inverted faces*, *upright tables*, and *inverted tables*, from *top* to *bottom*. *Left* grand-average ERP time courses according to conditions and groups. The P1 and N170 time periods are marked by *orange* and *green shadows*, respectively. *Middle* scalp topographies of the P1 component over the different conditions and groups. *Right* scalp topographies of the N170 component over different conditions and groups

EEG data were filtered using a low pass of 30 Hz (24 dB/oct) off-line. Epochs were face stimulus-locked and began 200 ms before face onset and ended 600 ms after face onset. Ocular artefacts were removed from EEGs using a regression procedure implemented in Neuroscan software (Scan 4.5). Trials exceeding the threshold of ±80 μV were excluded from further analysis. As a result, 14.3% of the epochs obtained from all participants were rejected. To test the possible accepted trial numbers difference between group, we run two t-tests of accepted trial numbers for pre-SAD (*M* = 51.35, *SE* = 0.99) vs. HC(*M* = 52.35, *SE* = 0.55), and post-SAD (*M* = 50.94, *SE* = 1.11) vs. HC. The results showed no group difference in trial numbers, all *t* < 1.009, all *p* > 0.33. Trials using the four conditions of interest (upright faces, inverted faces, upright tables, and inverted tables) were averaged, and a −200- to 0-ms baseline was used to perform a baseline correction (Fig. 2).

### ERP analysis

We first analysed group differences (HC vs. SAD) in ERPs in response to faces and objects before CBGT. The grand-averaged ERPs at PO7/PO8 and the corresponding topography maps of the N170 in the two groups are presented in Fig. 2. Based on previous literature [73] and visual inspection of the grand-averaged ERPs, we analysed the P1 and N170 over P5, P6, P7, P8, PO5, PO6, PO7, and PO8. The P1 amplitude was detected as the peak amplitude in the time window of 70–120 ms, while the N170 amplitude was detected as the peak amplitude in the time window of 120–200 ms. Since existing studies have suggested that the face inversion effect is associated with the slope between the P1 and N170 peaks [74], we also calculated the slope from P1 to N170 using the formula (P1_amplitude_ − N170_amplitude_/N170_latency_ − P1_latency_). Mean P1/N170 amplitude, peak latencies of P1/N170, and P1–N170 slope values were averaged across selected electrodes, and then entered into three hypothesis-driven testing analyses of variance (ANOVAs). There were 15 total ANOVAs conducted (3 ANOVAs for P1 amplitude, 3 ANOVAS for N170 amplitude, 3 ANOVAs for P1 latency, 3 ANOVAs for N170 latency, 3 ANOVAs for P1–N170 slope). First, a 4 (condition: upright faces vs. inverted faces vs. upright tables vs. inverted tables) × 2 (group: SAD vs. HC) ANOVA was conducted to test assess abnormalities in pre-therapy processing in the SAD and HC groups. An ANOVA was also conducted to examine the therapy effect on SAD for the different conditions, a 4 (condition: upright faces vs. inverted faces vs. upright tables vs. inverted tables) × 2 (time: pre-therapy SAD vs. post-therapy SAD). Lastly, a 4 (conditions: upright faces vs. inverted faces vs. upright tables vs. inverted tables) × 2 (time: HC pre-therapy vs. SAD post-therapy) ANOVA was conducted to examine whether CBGT “normalized” ERP patterns for the different conditions in patients with SAD post-therapy. Considering the slope test and the P1/N170 component analysis was not independent, we apply multiple comparison correction point of p < 0.025 across measure type (e.g. p < 0.025 for slope analyses for the P1 and N170 components, same for latency analyses, etc.). The reported degrees of freedom of the *F*-ratio were corrected using the Greenhouse–Geisser method when the sphericity assumption was violated. To investigate relationship between social anxiety symptom ERP component amplitude, we then run correlations between P1/N170 amplitude (P1-pre, N170-pre, P1-post, N170-post) to the anxiety symptoms(IAS-pre, IAS-post). Considering the unique connection is 4 in our initial analysis, the Bonferroni-corrected *p* value should be 0.05/4_(total number of comparisons)_ = 0.0125.

## Results

### Behavioural results

#### Overall behavioural performance

There were 60 targets (flowers) in each test, and all participants had equally good accuracy in target monitoring performance. The mean counts were 60.13, 60, 59.82, 59.88 for the SAD pre-therapy, HC, and SAD post-therapy groups, respectively. No differences were found between the HC and SAD pre-therapy groups, or between the SAD pre-therapy and SAD post-therapy groups, *p*s > 0.19.

#### Anxiety scores and treatment outcomes of CBGT

A t-test on the IAS scores of the SAD and HC groups revealed higher social anxiety in patients with SAD (mean [*M*] = 57.58, standard error [*SE*] = 1.27) than in HCs (*M* = 34.47, *SE* = 1.69) before the therapy, *t*_16_ = 8.67, *p* < 0.001. A t-test on the IAS scores of patients with SAD before and after therapy revealed significantly lower social anxiety after therapy (*M* = 38.59, *SE* = 2.29), *t*_16_ = 7.61, *p* < 0.001. The IAS scores of the patients with SAD post-therapy were not significantly different than those of the healthy controls, *t*_16_ = 1.32, *p* = 0.21.

The SAD group had higher BSPS scores (*M* = 45.12, *SE* = 2.79) than the HC group (*M* = 9.65, *SE* = 1.32) before the therapy, *t*_16_ = 10.11, *p* < 0.001. Changes in the BSPS score also suggested that social anxiety symptoms improved after therapy (*M* = 18.24, *SE* = 2.20), *t*_16_ = 10.67, *p* < 0.001. However, the BSPS score was still higher in patients with SAD post-therapy than in HCs, *t*_16_ = 3.31, *p* < 0.01.

### ERP results

#### Pre-CBT SAD vs. HC

##### P1 results

We carried out a repeated measures ANOVA on P1 amplitudes of patients with SAD pre-therapy vs. those of HCs. There was no significant group × condition interaction effect (*F*_1, 16_ = 1.63, *p* = 0.195, $\eta_{p}^{2}$ = 0.092), the group effect did not reach significance (*F*_1, 16_ = 3.58, *p* = 0.062, $\eta_{p}^{2}$ = 0.201). As shown in Fig. 2, the main effect of condition (*F*_3, 14_ = 9.82, *p* < 0.001, $\eta_{p}^{2}$ = 0.678) suggested that the inverted faces elicit larger P1 responses (*M* = 7.75 μV, *SE* = 0.53) than upright faces (*M* = 6.88 μV, *SE* = 0.49), upright tables (*M* = 6.45 μV, *SE* = 0.48), or inverted tables (*M* = 6.54 μV, *SE* = 0.47), all *p*s < 0.01 (Fig. 2). The other three conditions were not significant different from each other. An analysis of P1 latency in patients with SAD pre-therapy vs. HCs revealed a main effect of condition (*F*_3, 14_ = 12.54, *p* < 0.001, $\eta_{p}^{2}$ = 0.729), with upright faces leading to earlier P1 responses (*M* = 93.06 ms, *SE* = 1.85) than the other three conditions, *p*s < 0.05.

##### N170 results

A repeated measures ANOVA on the N170 amplitude in patients with SAD pre-therapy vs. HCs failed to find significant group effect (*F*_1, 16_ = 3.11, *p* = 0.097, $\eta_{p}^{2}$ = 0.097). However, a significant main effect of condition (*F*_3, 14_ = 68.00, *p* < 0.001, $\eta_{p}^{2}$ = 0.809) suggested that inverted faces elicit larger N170 responses (*M* = −11.29 μV, *SE* = 1.43) than upright faces (*M* = −10.38 μV, *SE* = 1.49), upright tables (*M* = −3.88 μV, *SE* = 1.05), or inverted tables (*M* = −3.42 μV, *SE* = 1.00), all *p*s < 0.05 (Fig. 2). An analysis of N170 latency in patients with SAD pre-therapy vs. HCs revealed a main effect of condition (*F*_3, 14_ = 18.48, *p* < 0.001, $\eta_{p}^{2}$ = 0.54), with upright faces eliciting earlier N170 responses (*M* = 145.06 ms, *SE* = 2.18) than the other three conditions, *p*s < 0.001.

##### Slope from P1 to N170

To better understand changes in the morphologies of the ERP waveforms, we analysed the slope from P1 to N170 [47]. The SAD group had a steeper change in this slope (*M* = 0.32, *SE* = 0.03) than the HC group (*M* = 0.23, *SE* = 0.02), *F*_1, 16_ = 7.02, *p* = 0.017, $\eta_{p}^{2}$ = 0.30. Furthermore, the main effect of condition suggested that the slope is larger in response to faces than to tables, *F*_3, 14_ = 16.47, *p* < 0.001, $\eta_{p}^{2}$ = 0.779.

#### Pre-CBT SAD vs. Post-CBT SAD

##### P1 results

A repeated measures ANOVA of P1 amplitudes in SAD patients pre-therapy vs. post-therapy revealed a significant time effect (*F*_1, 16_ = 17.40, *p* < 0.001, $\eta_{p}^{2}$ = 0.521), suggesting that P1 amplitudes are reduced in patients with SAD after therapy (*M* = 6.19 μV, *SE* = 0.52). There was also a significant condition effect (*F*_1, 16_ = 12.49, *p* < 0.001, $\eta_{p}^{2}$ = 0.438), suggesting that inverted faces (*M* = 7.98 μV, *SE* = 0.68) and upright faces (*M* = 7.57 μV, *SE* = 0.58) elicit larger P1 responses than upright (*M* = 6.09 μV, *SE* = 0.48) and inverted tables (*M* = 6.44 μV, *SE* = 0.58), *p*s < 0.003 (Fig. 2). However, analysis of P1 latency in patients with SAD pre- vs. post-therapy did not reveal any significant effects, all *F*s < 1.97, all *p*s > 0.18.

##### N170 results

An ANOVA of N170 amplitude in patients with SAD pre-therapy vs. post-therapy revealed a significant effect of condition (*F*_3,14_ = 45.34, *p* < 0.001, $\eta_{p}^{2}$ = 0.739), suggesting that both inverted (*M* = −11.92 μV, *SE* = 1.74) and upright faces (*M* = −10.65 μV, *SE* = 1.69) elicit larger N170 responses than upright tables (*M* = −5.38 μV, *SE* = 1.22) and inverted tables (*M* = −4.93 μV, *SE* = 1.18), all *p*s < 0.05. Interestingly, we found a significant interaction effect between time and condition, *F*_1, 16_ = 6.86, *p* < 0.01, $\eta_{p}^{2}$ = 0.30, suggesting that the reduced N170 effect after therapy only occurs for face stimuli. The simple effect for each condition pre vs. post therapy showed that inverted faces led to a reduction in N170 in SAD post-therapy (*M* = −13.11 μV, *SE* = 1.95) than pre-therapy (*M* = −10.79 μV, *SE* = 1.81), and the significance is approaching the correction point, *p* = 0.043 (Fig. 2). Moreover, the analysis of N170 latency in patients with SAD pre-therapy vs. post-therapy revealed a no significant effects, all *F*s < 3.88.

##### Slope from P1 to N170

**Fig. 3:**
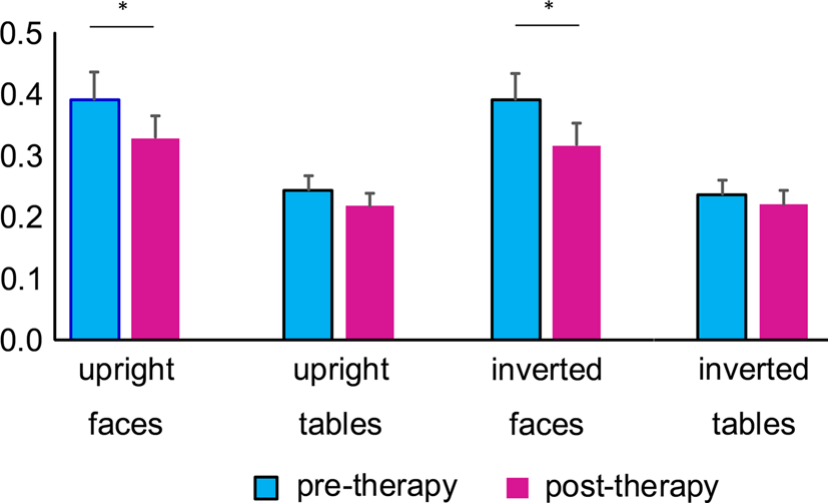
Slope from P1 to N170 for the *upright faces*, *upright tables*, *inverted faces* and *inverted tables*. The slope was calculated for SAD subject’s pre-therapy (*blue*) and post-therapy (*red*). *Error bars* denote standard error. *p < 0.05

Interestingly, when we analysed the slope from P1 to N170 in patients with SAD pre-therapy vs. post-therapy, we found a significant interaction between time and condition, *F*_1, 16_ = 6.33, *p* = 0.001, $\eta_{p}^{2}$ = 0.284. Further analysis indicated that the slope was generally flattened after therapy, simple effect analyses indicated the pre-post comparison was significant for upright faces (Pre: *M* = 0.39, *SE* = 0.05; Post: *M* = 0.33, *SE* = 0.04) and inverted faces (Pre: *M* = 0.39, *SE* = 0.04; Post: *M* = 0.32, *SE* = 0.04), *p*s < 0.013, but not to objects (Fig. 3).

#### Post-CBT SAD vs. HC

##### P1 results

ANOVA of P1 amplitudes in HCs pre-therapy vs. patients with SAD post-therapy only revealed a significant condition effect (*F*_1, 16_ = 5.84, *p* < 0.01, $\eta_{p}^{2}$ = 0.367), suggesting that inverted faces (*M* = 6.79 μV, *SE* = 0.49) elicit larger P1 responses than upright tables (*M* = 5.40 μV, *SE* = 0.42) and inverted tables (*M* = 5.89 μV, *SE* = 0.39), all *p*s < 0.008. No other group-related main effect or interaction effect was significant. Analysis of P1 latency in patients with SAD post-therapy compared to HCs showed a pattern that upright faces leading to earlier P1 responses, but was not significant, *p*s > 0.03 (main effect of condition*: F*_3, 14_ = 2.96, *p* = 0.041, $\eta_{p}^{2}$ = 0.156).

##### N170 results

ANOVA of N170 amplitudes in HCs pre-therapy vs. patients with SAD post-therapy only revealed a significant condition effect (*F*_1, 16_ = 47.89, *p* < 0.001, $\eta_{p}^{2}$ = 0.75), suggesting that inverted (*M* = −10.14 μV, *SE* = 1.35) and upright faces (*M* = −9.38 μV, *SE* = 1.39) elicit larger N170 responses than upright tables (*M* = −4.37 μV, *SE* = 0.88) and inverted tables (*M* = −3.73 μV, *SE* = 0.90), all *p*s < 0.025. No other group-related main effect or interaction effect was significant. Analysis of N170 latency in patients with SAD post-therapy compared to HCs only revealed a main effect of condition (*F*_3, 14_ = 4.38, *p* < 0.01, $\eta_{p}^{2}$ = 0.215), with upright faces leading to earlier N170 responses (*M* = 145.5 ms, *SE* = 2.42) than the inverted face conditions, *p* = 0.001.

##### Slope from P1 to N170

ANOVA of the P1–N170 slope in HCs pre-therapy compared to patients with SAD post-therapy only revealed a significant condition effect (*F*_1, 16_ = 23.79, *p* < 0.001, $\eta_{p}^{2}$ = 0.598), suggesting that inverted (*M* = 0.30, *SE* = 0.03) and upright faces (*M* = 0.31, *SE* = 0.03) had larger P1–N170 slopes than upright tables (*M* = 0.20, *SE* = 0.02) and inverted tables (*M* = 0.19, *SE* = 0.02), all *p*s < 0.001. No other group-related main effect or interaction effect was significant.

### Correlations between anxiety symptoms and ERP results

**Fig. 4:**
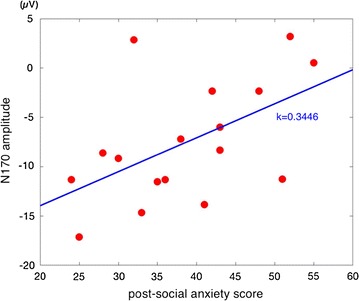
Mean amplitude of the N170 post-therapy as a function of post-therapy IAS score

To further confirm that higher social anxiety symptoms are correlated with enhanced early face-perception processing, bivariate correlation analyses were performed to examine the relationship between pre-therapy and post-therapy social anxiety scores and P1 and N170, respectively. We only found a significant positive correlation after correction between IAS score in patients with SAD post-therapy and post-therapy N170 amplitude, *r* = 0.675, *p* = 0.007 (Fig. 4). The correlation between pre-therapy social anxiety score and pre-therapy P1/N170 was not significant, *p*s > 0.23. Correlation analysis of changes in social anxiety scores from pre- to post-treatment and changes in P1/N170 did not reach significance, all *p*s > 0.27. No significant correlations were observed for P1.

## Discussion

The primary objective of this study was to determine whether social anxiety is associated with enhanced or reduced early neural responses to facial or non-facial stimuli. We also examined whether early perception abnormalities change after CBGT therapy. We assessed behavioural treatment effects on social anxiety symptoms after CBGT therapy using the IAS and the BSPS. Both IAS and BSPS scores were significantly reduced after CBGT. The IAS scores patients with SAD post-therapy were not different than those of HCs. Such results together indicated that social anxiety symptoms were attenuated after CBGT. Although CBGT has been shown to be effective in the treatment of SAD [49–53, 61]; Scaini et al. [54, 55, 75], only few preliminary studies on Chinese samples have been reported [76]. Our results thus provide further evidence for the effectiveness of CBGT for the treatment of SAD.

SAD is a mental disorder characterized by more negative attention to social cues and interpretation of social information. To examine whether individuals with social anxiety pay more early attention to faces than healthy subjects, we first compared data from patients with SAD pre-therapy to data from HCs. Our ERP results revealed a general facial inversion effect in both patients with SAD pre-therapy and HCs, such that inverted faces were associated with larger P1–N170 than other upward faces or non-face objects. Our results are consistent with those of previous studies reporting increased P1 or N170 in response to inverted faces (i.e., facial inversion effect), which mainly reflects the need for configurational processing of faces [31, 44, 77]. The facial inversion effect did not differ between patients with SAD and normal controls in our study, indicating that SAD does not lead to facial stimulus-related specific enhancement processing. However, we found a general trend for increases in the P1–N170 component in response to all stimuli in patients with SAD relative to controls.

The early visual P1 component is an early index of the low-level features of a stimulus and is modulated by emotion or salience [78, 79]. The P1 enhancement effect is also found in response to non-emotional stimuli when the location captures an individual’s attention [80] or when there is increased attention to a threat [81]. These findings provide strong evidence for the association between early selective attention and P1. Our results replicated the findings of several previous studies reporting larger P1 for faces than for objects [82, 83]. P1 responses to faces are also faster, which may reflect the early selective attention to social information. We found a trend for larger P1 responses to both faces and tables in individuals with SAD and HCs. This may indicate a common pattern of greater early attention to both faces and objects in individuals with SAD.

The sensitivity of N170 to the inversion of face stimuli has been observed in many previous studies [31, 45]. This reflects a holistic processing mechanism for faces. Our prior ERP study indicated that N170 abnormalities in SAD are associated with response bias [8], and that poor subjective discrimination and recognition ability for faces may stem from abnormal face perception or abnormal attention strategies. However, we did not find significant N170 amplitude group difference in our results.

The above findings seemingly do not support the hypothesis of early hypervigilance to facial stimuli in SAD, as the hypothesis states that socially anxious individuals overtly attend to initial socially relevant cues (e.g., faces) [84]. In other words, the above findings are inconsistent with the hypothesis that selective face processing might be impaired in SAD [8, 28, 85]. At least two interpretations of the lack of a selective attention effect to faces may exist. First, it is possible that previous studies of patients with SAD used different emotional faces [31, 86, 87], see [88], for a review). There is thus no direct evidence that patients with SAD have abnormalities in neutral face processing and not in object processing. An alternative explanation is that the task used in the current study is implicit (task-irrelevant), and that the attention of the participant is focused more on the target stimulus (i.e., flower) in all groups. In this case, both faces and objects are less attended to. If the latter is true, additional studies are required to elucidate the interaction between facial/object attention and social anxiety during explicit attention tasks.

Interestingly, we found a general decrease in the P1–N170 component in patients with SAD after the CBGT therapy sessions. We specifically observed smaller N170 responses in response to face stimuli. Similar changes from pre- to post-treatment change were also partly observed in previous studies of the treatment effects of CBT [66, 67], see [89], for a meta-analysis). For instance, Miskovic et al. [67] have reported frontal alpha EEG asymmetry changes from relatively greater right to relatively greater left frontal alpha EEGs in participants. To our knowledge, this is the first report of a relationship between CBT and ERP responses to faces. As stated above, the early face processing in our task was investigated implicitly (task-irrelevant), as the explicit task was flower-counting. Therefore, CBGT leads to smaller P1–N170 responses to faces even in implicit tests. The current results thus imply that CBGT modulates early face attention and attenuates hypervigilance to social cues even when there is low global attention to the stimuli.

It has been proposed that the facial inversion effect is reflected in the slope between P1 and N170, which combines the peak to peak change in amplitude and the peak to peak change in latency [74, 90]. Although there was no significant group difference on P1 or N170, the SAD abnormal effect occurs in P1–N170 slope. That is, the SAD group showed steeper slope than HC subjects, which may reflect the relative higher early face/object processing sensitivity in SAD. Therefore, it is possible that the P1–N170 slope could serve as a clinical index to determine abnormality in SAD.

Importantly, the CBGT treatment not only weakens anxiety symptoms, but also reduces face-related early components and the P1–N170 slope, especially for face related stimuli. According the cognitive-motivation account of anxiety, anxiety influences the appraisal of stimulus threat value [91]. Compared with objects, facial stimuli are more associated with less threat after the treatment, which could also be changed with the anxiety level [92]. Such treatment effect on face-related early components and the P1–N170 slope in patients with SAD may also be in agreement with our hypothesis that treatment could changes the selective processing in facial stimulus, due to temporal-occipital N170 was more sensitive to holistic processing of faces[93]. Due to the lack of spatial information in the EEG study, further brain imaging studies should be considered to better understand the neural mechanisms underlying such treatment effects in socially anxious individuals.

Although we did not conduct a control therapy procedure in the HCs, the correlation between social anxiety scores after therapy and the corresponding N170 amplitude may provide further support for the relationship between N170 and social anxiety symptoms. In the current study, there was no correlation between symptom improvement and ERP amplitude, which may due to the relatively small sample size. To assess the clinical implications of our work, it would be beneficial to study the possible correlation between clinical social anxiety symptoms and the corresponding ERP amplitude or slope.

Several limitations of this study should be noted. First, the sample size was relatively small due to difficulties with the experimental design (1-year follow-up). Together with the fact that we had a larger proportion of women in our sample, this may have led to reduced statistical power and generalized inference in the current study. Second, our study was limited to neutral face and object conditions in an implicit task. More conditions and tasks should be considered to investigate treatment effects. Another limitation of our study was that we did not carry out control treatment in the HC group due to difficulties with the treatment in HCs (treatment motivation control). It would have been helpful to have a third group in the study that included individuals with SAD who did not complete CBGT to ensure that our findings are in fact related to the intervention and not just familiarity with the stimuli in the task. An additional limitation of the current study is the fact that individuals need to use working memory throughout the task to maintain and manipulate the number of previously viewed flowers. This poses a significant limitation, which may have affected the ERP signal in response to faces in individuals with SAD, as the core element of SAD is fear of negative evaluation [94, 95]. In other words, the subjects with SAD might have been even more motivated to remember the numbers of flowers due to fear of negative evaluation. This would have led to less processing of stimuli in the second viewing task (the post-therapy test), especially for facial stimuli. Further studies with more controls and varied materials should be considered to explore this research question.

## Conclusions

In conclusion, data from this study provide support for early enhanced attention and rapid reactions to both faces and objects in patients with SAD. Administration of CBGT can “normalize” this early hyperactivity, especially for facial stimuli in socially anxious individuals who exhibit aberrant P1–N170 responses. Our data provide initial evidence for an implicit processing of faces and objects in individuals with SAD and for brain-based improvements following therapy. It is hoped that the current results encourage further examination of potentially complex conditions and brain imaging methods to carry out brain-based SAD assessments and to predict treatment effects.
